# Mysterious long noncoding RNAs and their relationships to human disease

**DOI:** 10.3389/fmolb.2022.950408

**Published:** 2022-11-02

**Authors:** Wenchao Li, Yang Yang Wang, Lifei Xiao, Jiangwei Ding, Lei Wang, Feng Wang, Tao Sun

**Affiliations:** ^1^ Ningxia Key Laboratory of Craniocerebral Disease, Incubation Base of National Key Laboratory, Ningxia Medical University, Yinchuan, China; ^2^ The First Affiliated Hospital of Xinxiang Medical University, Xinxiang, China; ^3^ Zhejiang University School of Medicine, Hangzhou, China

**Keywords:** noncoding RNA, tumors, nervous system, disease, epigenetics

## Abstract

Increasingly studies have shown that the formation mechanism of many human diseases is very complex, which is determined by environmental factors and genetic factors rather than fully following Mendel’s genetic law of inheritance. Long non-coding RNA (lncRNA) is a class of endogenous non-protein coding RNA with a length greater than 200 nt, which has attracted much attention in recent years. Studies have shown that lncRNAs have a wide range of biological functions, such as roles in gene imprinting, cell cycle progression, apoptosis, senescence, cell differentiation, and stress responses, and that they regulate the life processes of mammals at various levels, such as epigenetic transcription, processing, modification, transport, translation and degradation. Analyzing the characteristics of lncRNAs and revealing their internal roles can not only deepen our understanding of human physiological and pathological processes, but also provide new ideas and solutions for the diagnosis, prevention and treatment of some diseases. This article mainly reviews the biological characteristics of lncRNAs and their relationship with some diseases, so as to provide references for the related research of lncRNAs.

## Introduction

Ribose nucleic acid (RNA) is a single strand formed by transcribing a deoxyribonucleic acid (DNA) strand as a template according to the principle of complementary base pairing. In recent years, with the rapid development and improvement of next-generation high-throughput sequencing technologies, our understanding of the field of transcription has been expanded. An important finding is that although nearly 80% of the genome can be transcribed, only approximately 2% of mammalian genomes consist of protein-coding genes, most of the rest of the genome sequence only produces a large amount of noncoding RNA (ncRNA). These RNAs can only be successfully transcribed and lack the ability to directly encode proteins, only a few lncRNAs can encode small molecular peptides. Previously, researchers believed that non-coding RNA, as a kind of gene “garbage”, had no special biological effect (Wang and Chang, 2011). These non-coding RNAs are roughly divided into two classes, depending on the length of the transcript. That is, small noncoding RNA (snRNA) with a length less than 200 nt and long non-coding RNA (lncRNA) with a length greater than 200 nt. Interesting, lncRNAs are the largest component of non-coding transcripts in mammals, accounting for about 80% (Vennin and Adriaenssens, 2018).

With further research, scholars found that lncRNAs can play the role of “regulatory factors” to participate in various levels of transcription, post-transcription and translation, and also participate in various biological processes, such as embryo development invasion, apoptosis, metastasis and angiogenesis (Renoux and Todd, 2012; Mazar et al., 2014). In cancer, metabolic diseases and nervous system diseases, lncRNA expression profile is also changed, and dysregulation of lncRNA expression is closely related to the occurrence and development of diseases. Moreover, studies have revealed that the expression of lncRNAs is more cell-specific than that of protein-coding genes. A considerable number of lncRNAs showed 3′ polyadenylation and 5′ caprization, with multiple exons, and showed transcriptional activation activity similar to mRNA (Cabili et al., 2011). The biological characteristics, functions, and their relationship with diseases of lncRNAs have become a research hotspot.

## Biological characteristics of long non-coding RNAs

### Classification of long non-coding RNAs

**FIGURE 1:**
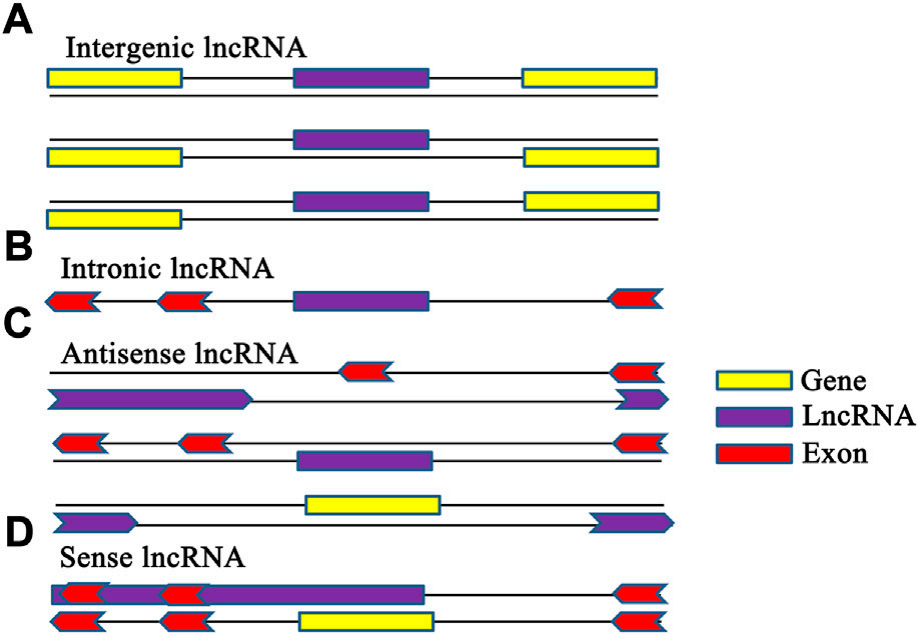
Classification of lncRNAs. Panel **(A)**: Intergenic lncRNA was transcribed intergenically from both strands. Panel **(B)**: intronic lncRNA, transcribed entirely from introns of protein-coding genes. Panel **(C)**: antisense lncRNA, transcribed from the antisense strand of protein-coding genes, overlapping with exonic or intronic regions or covering the entire protein-coding sequence through an intron. Panel **(D)**: Sense lncRNA was transcribed from the sense strand of protein-coding genes and contain exons from protein-coding genes, overlapping with part of protein-coding genes or covering the entire sequence of a protein-coding gene through an intron.

In order to carry out more in-depth research on lncRNAs and understand their functions and mechanisms, researchers divided lncRNAs into intronic lncRNAs, intergenic lncRNAs, sense lncRNAs and antisense lncRNAs four categories according to their location and background in the genome (Ma et al., 2013). Intergenic lncRNAs may be located more than 10 kb from any nearby protein-coding locus. But, intronic lncRNAs originate from long introns that are transcribed from the same strand as the associated protein-coding genes. Studies suggest that intergenic lncRNAs and intronic lncRNAs may play regulatory roles through different transcriptional activation mechanisms, and may have different poly (A) modification and expression activities at different locations of cells (Cheng et al., 2005; Prensner et al., 2011). Antisense lncRNAs are lncRNAs that are transcribed from the antisense strand of a gene locus and overlap with the RNA transcribed from the sense strand. In contrast, sense lncRNAs are lncRNAs that contain a protein coding gene and are transcribed in the same direction as that gene (Figure 1).

### The mode of action of long non-coding RNAs

**FIGURE 2:**
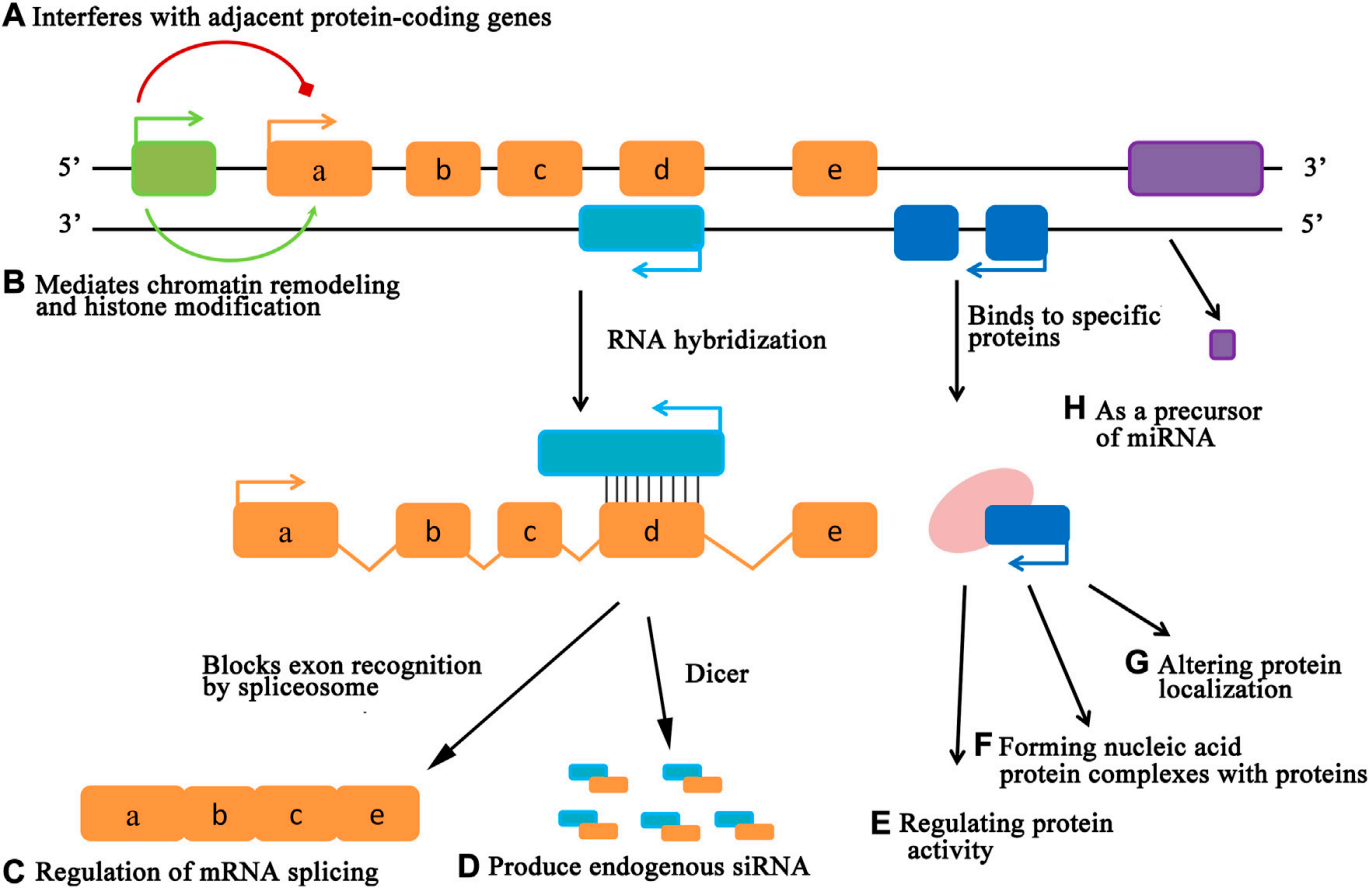
The mode of action of lncRNAs. LncRNAs can regulate gene expression levels at various levels, including epigenetic regulation, transcriptional regulation, and post-transcriptional regulation.

As another new field in molecular biology, with the in-depth study of lncRNAs, researchers have found that the main function of lncRNA may be to combine with DNA, RNA and protein to play a regulatory role (Zhu et al., 2013). As a regulatory molecule of gene expression, the mode of action of lncRNAs can be roughly divided into three aspects. One approach is that lncRNAs affect gene expression by regulating chromatin epigenetic modifications (Figure 2B). In 2013, Di ruscio et al. (2013) showed that the lncRNA derived from the CCAAT-enhancer binding protein-alpha (CEBPA) gene can interact with the DNA methyltransferase DNMT1 to regulate the methylation level at the CEBPA gene locus, thereby promoting gene expression.

As a molecular scaffold or bridge, regulating the related chromatin modifying enzymes in the transcription process, thereby changing the level of gene expression is another mode of action of lncRNAs (Figures 2A,D,E). The non-coding RNA snRNA7SK, the guardian of transcriptional termination and bidirectional transcription in embryonic stem cells, acts as a scaffold for the protein complex formed by HEXIM1, HEXIM2, LARP7 and P-TEFb. The formation of this complex causes the loss of P-TEFb kinase activity and affects the transcriptional activity of related genes (Castelo-branco et al., 2013).

The regulation of gene expression by lncRNA in the post-transcription process is mainly achieved by the level regulation of mRNA and miRNA (Figures 2C,F–H). Cesana et al. (2011) found in their study that LINCMD1 could bind to corresponding miRNA, thus blocking the binding of target gene and miRNA, so as to lose its transcriptional inhibition of target gene.

## Relationship between long non-coding RNA and disease

### Tumors

At present, the most in-depth research on the function of lncRNAs was their role in tumor diseases. A large number of studies have shown that lncRNAs play an extremely important role in the occurrence and development of tumors (Harries, 2012; Kitagawa et al., 2012). Interestingly, lncRNAs not only promote tumor formation as a proto-oncogene, but also inhibit tumor cell proliferation and migration as a tumor suppressor gene. The occurrence and development of many cancers are accompanied by abnormal expression of lncRNAs. Zhu et al. analyzed the bladder cancer pathological tissues by microarray showed that 3324 lncRNAs were abnormally express, including 22 lncRNAs were significantly up-regulated and 88 lncRNAs were significantly down-regulated (≥8-fold change) in the tumor group compared with the controls. Further verification experiments found that the expression changes of lncRNA TNXA, CTA-134P22.2, CTC-276P9.1 and KRT19P3 were highly consistent with microarray data. They also observed that down-regulated lncRNAs were more common than up-regulated lncRNAs (Zhu et al., 2014a). In addition, studies have confirmed that lncRNA H19 is highly expressed in bladder cancer patients, and the expression level was 3 times higher than that in normal patients. Then *in vivo* and *in vitro* experiments have further confirmed that up-regulation of lncRNA H19 can accelerate the metastasis of bladder cancer cells. The researchers believe that lncRNA H19 can first bind to enhancer of zeste homolog 2 (EZH2), then activate Wnt/β-catenin, and down-regulate E-cadherin (E-cad). Therefore, lncRNA H19 may promote bladder cancer metastasis by combining with EZH2 and inhibiting the expression of E-cad (Luo et al., 2013).

It is worth noting that, in addition to bladder cancer, the researchers found that lncRNA H19 also plays a regulatory function in prostate cancer. LncRNA H19-mir675 axis may inhibit the metastasis of prostate cancer by transforming growth factor beta induced protein (TGFBI) (Zhu et al., 2014b). Among them, TGFBI is closely associated with cancer metastasis, and miR675 can directly bind to the 3′UTR of TGFBI-mRNA to inhibit the normal translation of TGFBI. HOTAIRM1, as lincRNA (long intergenic noncoding RNA), was found to be highly specifically expressed in mature myeloid cells. The barretts esophagus (BE) is considered the precancerous lesion of the esophageal adenocarcinoma (EAC), which results in 80 percent of esophageal adenocarcinoma. Previous studies have reported that lncRNA AFAP1-AS1 is highly expressed in esophageal adenocarcinoma and Barrett esophageal tissues. Targeted silencing of lncRNA AFAP1-AS1 can inhibit the differentiation of EAC, promote its apoptosis, and inhibit the invasion and metastasis of tumor cells without affecting the expression of AFAP1 protein (Wu et al., 2013).

Studies related to urological tumors have shown that lncRNA urothelialcarcinorna associated 1 (UCA1) can promote the proliferation of bladder cancer cells BLS-211 by regulating genes, and can enhance its drug resistance (Wang et al., 2008). A study conducted by Silva et al. (2011) shown long stress induced non-coding transcripts 5 (LSINCT5), is highly expressed in breast and ovarian cancer. LSINCT5 has greater than ten-fold increased expression in all cancer cell lines tested as compared to normal cell lines from the same tissues. Several breast cancer cell lines had 30-fold higher expression of LSINCT5 than in HMEC, including MDA468, T47D and BT474 cells. Targeted knockout of lncRNA LSINCT5 significantly inhibited the proliferation of cancer cells. Other studies have found that in breast cancer, the expression level of growth rest gene transcript 5 (Gas5) tends to reduce to 34% of those of adjacent normal tissue, while the Gas5 can induce cell apoptosis by regulating the target gene and play the function of tumor suppressor gene (Mourtada-maarabouni et al., 2009). In addition, Askarian-amiri et al. (2011) confirmed zinc finger antisense 1 (ZFAS1) was also significantly down-regulated in breast cancer. The result shows ZFAS1 expression is decreased (2.0-fold) in ductal carcinoma relative to normal epithelial cells. Taken together with the effects of ZFAS1 knockdown on mammary epithelial cell proliferation and differentiation, their results suggest ZFAS1 as a novel human tumor suppressor gene in breast cancer and that its dysregulation may be useful as a marker for breast cancer.

### Neurodegenerative and psychiatric diseases

With the deepening of research, it has been gradually recognized that there are a large number of specifically expressed lncRNAs in the mammalian brain, and the occurrence and development of many neurological diseases are often accompanied by abnormal expression of some lncRNAs (Knauss and Sun, 2013). Alzheimer’s disease (AD) is one of the most common neurological diseases. Studies demonstrate that AD is caused by the abnormally high expression of amyloid-β (Aβ) in the brain, and then form senile plaque (SP), while Aβ is the product after the splicing processing of amyloidprecursor protein (APP) by secretase. Β-secretase1 (BACE1) is one of the secretase enzymes that splicing APP, which plays a key role not only in the production of Aβ and but also in the aggregation of Aβ.

It has been reported that the antisense lncRNA BACE1AS of BACE1 plays a very important role in the occurrence and development of AD. Under stress conditions, BACE1AS can form complex with BACE1-mRNA to increase the stability of the latter and prevent its degradation, thus promoting the aggregation of β -amyloid protein. Subsequent experimental studies also found that BACE1AS was highly expressed in AD patients and BACE1 transgenic mice. In addition, studies have found that amyloid beta protein 142 (Aβ142) can inhibit the differentiation of SH-SY5Y cell, induce the expression of APP-related factors and the formation of senile plaques. In AD model group, the expression of Aβ142 and Aβ140 protein and mRNA were up-regulated, accompanied by the down-regulation of Ki67 expression. It was also confirmed that exogenous Aβ142 not only promoted the expression of BACE1, but also promoted the expression of lncRNA BACE1AS. LncRNA BACE1AS can increase the stability of BACE1mRNA. In the subsequent reverse validation test, targeted down-regulation of lncRNA BACE1AS expression in SH-SY5Y cells attenuated the ability of BACE1 to cleave APP and slowed the formation of senile plaques in SP AD SH-SY5Y model (Faghihi and Wahlestedt, 2009; Liu et al., 2014). Recent studies have reported that the occurrence and development of AD is also related to the variable expression and abnormal location of BC200 RNA. Mus et al. (2007) found that BC200 was significantly up-regulated in AD brains, and this up-regulation in AD was specific to brain areas that are involved, BC200 levels are specifically elevated in area 9, which is involved in the AD, but not in area 17, which is generally not. Not only that, the researchers found relative BC200 levels in those areas increased in parallel with the progression of AD.

LncRNAs are also involved in the regulation of psychiatric diseases. The occurrence and development of related diseases such as major depression, autism spectrum disorder, schizophrenia, affective schizophrenia and bipolar disorder are closely related to the abnormal expression of disorder in schizophrenia-1 (DISC1) (Chubb et al., 2008). LncRNA DISC2 regulates DISC1, and lncRNA DISC2 may be a potential target for the treatment of psychiatric disorders. In addition, Tamura et al. (2007) have reported the possibility that epigenetic aberration from the normal DNA methylation status of RELN may confer susceptibility to psychiatric disorders.

### Endocrine disease

Diabetes is a metabolic disease caused by a variety of factors. Its main clinical features are chronic hyperglycemia and metabolic disorders of sugar, fat and protein caused by impaired insulin secretion or defective insulin action. The World Health Organization classifies diabetes into four broad categories: I-diabetes, II-diabetes, gestational diabetes and other types of diabetes.

The pathogenesis of different types of diabetes is different, but most of them are pancreatic islet β-cell dysfunction, unable to secrete enough insulin, resulting in hyperglycemia. At present, it has been confirmed that miRNA plays an important role in the occurrence and development of diabetes. However, only a few studies have reported the role of lncRNAs in the occurrence and development of diabetes. For example, the antisense transcript IGF2AS of Insulin likegrowth factor 2 (IGF2) and the antisense transcript lncRNA PINK1 of PTENinduced putative kinase 1 (PINK1) lost on chromosome X. High concentration of glucose can stimulate up-regulation of IGF2AS expression in pancreatic islet β-cell, suggesting that IGF2AS expression may be correlated with blood glucose concentration (Mutskov and FELSENFELD, 2009). PINK1 can be activated by PTEN, which is an important inhibitor of insulin signaling pathway. In addition, array analysis performed by Scheele et al. (2007a); Scheele et al. (2007b) demonstrated a reduction in muscle PINK1 expression due to reduced activity of participants in the experiment; then their qRT-PCR data confirmed that PINK1 was 40% reduced following 5 weeks of inactivity in healthy volunteers. In contrast, natural antisense PINK1 (naPINK1) tended to be up-regulated (50%). In general, lncRNA-related studies are still in the initial stage, and there are fewer reports on the relationship between lncRNA and diabetes, which requires a lot of research work by various research teams in the later stage.

### Substance use disorder

Substance use disorder is an uncontrollable, chronic and recurrent brain disease characterized by compulsive drug seeking and continuous craving, which causes serious physical and psychological harm to users. In addition, drug use disorder will lead to an increase in social crime rate and the spread of HIV, hepatitis and other related infectious diseases, which has become a global public health problem (Merz, 2018; Heikkilä et al., 2021). The formation mechanism of substance use disorder is very complex. In the process of continuous exploration and research, the epigenetic mechanism has attracted more and more attention from experts in different fields. Epigenetics refers to changes in gene expression through DNA methylation, histone modification, chromatin remodeling, and non-coding RNA regulation without changes in genetic information and DNA sequence (Goldman et al., 2005; Godino et al., 2015). Previous studies have shown that lncRNAs can regulate gene expression through epigenetic mechanisms, play an important role in the formation of synaptic plasticity, and then promote the formation of drug use disorder. In addition, researchers believe that addictive drugs can alter gene expression in brain tissues. In the hippocampus of cocaine-induced conditioned place preference (CPP), 214 transcripts were altered, and 151 genes were increased significantly. These genes belong to several functional classes including transcription, translation and protein synthesis, signal transduction, protein kinases/phosphatases, metabolic enzymes and cytoskeleton organization. Subsequently, 39 genes were found to be significantly altered in the prefrontal cortex (PFC), of which 22 genes were transcriptionally increased and 17 genes were transcriptionally decreased. Their data support the possibility that genes changes in the hippocampus and cortex might participate in the formation and of memory patterns induced by cocaine. In addition, CM156, as α -receptor antagonist, can reduce cocaine-induced CPP formation. Meanwhile, CM156 can also reverse the expression trend of some cocaine-induced transcripts in rat brain, including MATAL1, suggesting that MATAL1 may play a certain role in regulating gene expression under cocaine exposure and act through α-receptor (Kalivas and Brien, 2008; Krasnova et al., 2008; Xu et al., 2012).

Nucleus accumbens were extracted from victims of cocaine and methamphetamine use disorders. Transcriptome study found that the two addictive drugs had effects on the transcription of related genes, and the affected genes rarely overlapped. Even about half of the overlapping genes are regulated in opposite trend (Albertson et al., 2006), which suggests the possibility that cocaine and methamphetamine have different mechanisms of action in the brain. In subsequent studies, it was found that this sequence transcribed lncRNA MIAT (myocardial infarction associated transcript) (Ishii et al., 2006). At present, MIAT has been confirmed to be involved in oligodendrocyte formation and nuclear matrix formation (Mercer et al., 2010). Subsequently, Michelhaugh et al. (2011) used affymetrix microarray to study the expression changes of lncRNA in heroin abusers’ brain tissues, and found that the expressions of MIAT, MEG3 and NEAT1/2 were up-regulated in nucleus accumbens. A genome-wide scan study in Caucasians also showed that MEG3 may be associated with heroin addiction (Nielsen et al., 2008). Moreover, MEG3 is expressed in the nucleus as a maternal imprint and exists in different subsets of neuronal cells, which is related to early neurogenesis. Knockout of MEG3 in the mouse brain resulted in altered expression of angiogenic genes and increased microvascular formation. The expression changes and functions of these lncRNAs indicate that relevant lncRNAs may play an important role in post-transcriptional regulation and neural adaptation after drug use disorder.

Recently, the effects of alcohol on lncRNA expression in the nervous system have also attracted attention. MALAT1 transcription level was significantly up-regulated in the cerebellum of patients with chronic alcohol use disorder, as well as in the hippocampus (1.8-fold) and brain stem (1.5-fold), but not in the frontal lobe and motor cortex (Kryger et al., 2012). In later animal experiments, the researchers found that the expression of lncRNAs in the brain did not change significantly during acute exposure to alcohol in rats. However, MALAT1 was significantly up-regulated in rat cortex during alcohol withdrawal. MALAT1 can bind to SR splicing proteins to regulate alternative splicing of precursor mRNA (Tripathi et al., 2010). These results suggest that alcohol-mediated up-regulation of MALAT1 may play an important role in the expression of different isoforms or variants of neurotransmitter receptors and ion channel-related proteins.

## Summary and prospect

LncRNAs are a very important part of eukaryotic transcripts. As the main body of ncRNAs, lncRNAs can regulate more than 70% of gene expression and play a very important role in physiological and pathological processes of the body. At present, the study of lncRNAs has become a new direction in the field of RNA, but it is still in the primary phase. The natures, structures and functions of many lncRNAs have not been clarified. Although existing researches suggest that lncRNAs play an important role in a variety of systemic diseases, there are still many basic and application problems to be solved, and their characteristics and regulation mechanisms need to be further clarified.

Human life activities depend on complex regulatory networks among DNA, RNA and proteins in the body. Therefore, future research will tend to study the human life process from a holistic and systemic perspective. Studying the expression of proteins, DNA and RNA in specific cells in different time and space and their interrelationships is delicate. Accurately studying the regulatory network of proteins, DNA and RNA in specific tissues or even in specific space-time is one of the core issues to clearly reveal human life activities. In current review, we summarize the research progress of lncRNAs in tumors, neurodegenerative and psychiatric diseases, endocrine disease and substance use disorder. Among them, some lncRNAs may well serve as biomarkers for diagnosing disease, but their accuracy and stability need to be carefully evaluated in preclinical research stage. In addition, the potential role of lncRNA in other diseases also needs to be explored and summarized. It is believed that with the in-depth study of lncRNAs by scholars, it will not only deepen human’s further understanding of the regulatory network of disease occurrence and development, but also provide a basis for disease diagnosis and new therapeutic targets and approaches.
